# Forecast of Fine Particles in Chengdu under Autumn–Winter Synoptic Conditions

**DOI:** 10.3390/toxics11090777

**Published:** 2023-09-13

**Authors:** Jingchao Yang, Ge Wang, Chao Zhang

**Affiliations:** 1 Institute of Plateau Meteorology, China Meteorological Administration, Chengdu 610072, China; bfxyangning@sina.com; 2 Heavy Rain and Drought-Flood Disasters in Plateau and Basin Key Laboratory of Sichuan Province, Chengdu 610072, China; 3 Department of Atmospheric Science, School of Environmental Studies, China University of Geosciences, Wuhan 430074, China

**Keywords:** Chengdu, CMAQ, objective synoptic weather classification, PM_2.5_, WRF

## Abstract

We conducted an evaluation of the impact of meteorological factor forecasts on the prediction of fine particles in Chengdu, China, during autumn and winter, utilizing the European Cooperation in Science and Technology (COST)733 objective weather classification software and the Community Multiscale Air Quality model. This analysis was performed under four prevailing weather patterns. Fine particle pollution tended to occur under high-pressure rear, homogeneous-pressure, and low-pressure conditions; by contrast, fine particle concentrations were lower under high-pressure bottom conditions. The forecasts of fine particle concentrations were more accurate under high-pressure bottom conditions than under high-pressure rear and homogeneous-pressure conditions. Moreover, under all conditions, the 24 h forecast of fine particle concentrations were more accurate than the 48 and 72 h forecasts. Regarding meteorological factors, forecasts of 2 m relative humidity and 10 m wind speed were more accurate under high-pressure bottom conditions than high-pressure rear and homogeneous-pressure conditions. Moreover, 2 m relative humidity and 10 m wind speed were important factors for forecasting fine particles, whereas 2 m air temperature was not. Finally, the 24 h forecasts of meteorological factors were more accurate than the 48 and 72 h forecasts, consistent with the forecasting of fine particles.

## 1. Introduction

Various synoptic weather patterns affect fine particle transmission and diffusion in Chengdu, Sichuan Province, China, during autumn and winter [1,2]. The suspended fine particles that affect human health are mainly located near the surface, and their transport and diffusion are mainly affected by near-surface meteorological factors under different weather patterns [3,4]. Therefore, fine particle forecasting is directly affected by the forecasting of near-surface meteorological factors [5,6]. Hence, comprehending the impact of near-surface meteorological factor predictions on the estimation of fine particle concentrations in air quality models is crucial, considering diverse weather patterns, air quality indices, and temporal intervals.

Objective synoptic weather classification is based on cluster analysis of geopotential height and wind field datasets, and can be used to classify synoptic data. Many studies on fine particle pollution have applied this approach to analyze the impact of meteorological conditions. For instance, several studies on regional wind fields using objective synoptic weather classification reported that recirculation and stagnant wind fields provide conditions favorable for the development of fine particle pollution [7]. This method can also reveal how meteorological conditions contribute to regional fine particle pollution [8]. For instance, weakened airflows resulting from climate change can increase fine particle concentrations [9], which was also predicted using the EC-Earth climate model [10]. Additionally, aerosol optical depth and fine particle concentrations influence weather patterns [11].

The boundary layer structures associated with fine particle pollution differ among weather patterns [12]; thus, objective weather classification can be used to study both fine particle and ozone control. For example, in northeast China and the Poyang and Dongting Lakes areas, ozone pollution is dominated by weather patterns, whereas fine particles are mainly controlled by human activities [13,14]. Fine particle concentrations differ from ozone concentrations under identical weather patterns [15]. Typically, ozone pollution occurs under low-pressure, high-temperature, and high-radiation conditions [16]. In addition, objective synoptic weather classification has been employed to analyze dust pollution, pollen pollution, and the urban heat island effect under different weather conditions. For instance, in southern Beijing, strong heat island effects occur under weak high-pressure systems [17]. Moreover, during summer in the Iberian Peninsula, dust pollution tends to occur under southerly winds [18], while in Poland warm and dry anomalous anticyclonic systems may contribute to pollen pollution [19].

The Community Multiscale Air Quality (CMAQ) model is the most commonly used model to forecast regional atmospheric pollution, including fine particle concentrations. The CMAQ model has been used to analyze forecasts of fine particle concentrations. For instance, various studies have examined the direct impact of meteorological factor forecasts on the prediction of atmospheric pollutants [20], the utilization of micro-pulse lidar observations [21], the incorporation of Global Positioning System Zenith Total Delay data [22], and the implementation of an urban canopy parameterization scheme [23] to enhance the accuracy of fine particle forecasting using the CMAQ model. Researchers have also used machine learning [24], four-dimensional variational assimilation [25], and Kalman filter [26] methods to optimize fine particle forecasts by the CMAQ model. Also, the CMAQ model has been used to analyze the impact of changes in meteorological conditions on the regional transmission of fine particles [27,28], thereby facilitating policy-making related to the control of regional fine particulate emissions [29]. Finally, the CMAQ model has been used to identify the relative importance of emission sources, road sources, and regional transmission in regional air pollution [30], and to help predict future emission scenarios and trends of fine particles [31]. Overall, studies have focused on the impacts of different weather patterns on atmospheric pollutant emissions and distributions using the CMAQ model. Comparatively few studies have examined fine particle forecast accuracy using objective synoptic weather classification methods.

In the present study, the daily sea level pressure and 10 m wind fields in Chengdu, China, during autumn and winter in the period 2018–2022 were classified into dominant weather patterns based on the COST733 weather classification software. Subsequently, we conducted an analysis on the effectiveness of the CMAQ model in simulating fine particles from 1 November 2021 to 28 February 2022, while also assessing the influence of meteorological factor predictions on the forecast accuracy of fine particles.

## 2. Materials and Methods

### 2.1. Objective Synoptic Weather Classification

Using the objective synoptic weather classification software developed by the European Union COST733 Project, daily sea level pressure and 10 m wind fields, obtained from the fifth-generation reanalysis (ERA5) product provided by the European Center for Medium-Range Numerical Weather Forecasting, were decomposed into several components using principal component (PC) analysis. Components were sorted based on their eigenvalues. Components explaining large amounts of variance were selected and defined as PCs. The number of PCs should be less than or equal to the number of observation days. The cumulative variance explained by the selected PCs exceeded 85%. The selected PCs were obliquely rotated and classified into several groups representing weather patterns according to the magnitudes of the component loadings [32,33].

### 2.2. Model Parameters

**Figure 1:**
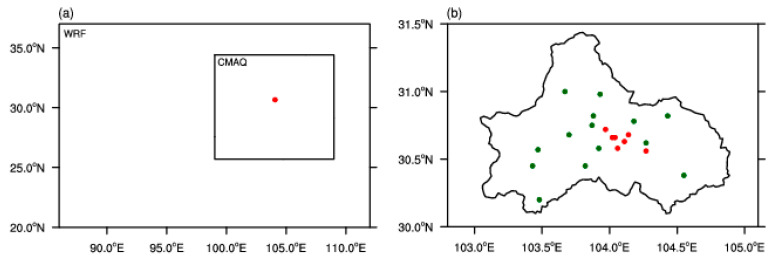
(**a**) The domains of the WRF and CMAQ models. The red dot indicates the position of Chengdu, China; (**b**) spatial distributions of environmental monitoring stations (red dots) and meteorological stations (green dots) in Chengdu.

In the Weather Research and Forecasting (WRF) model, the forecast area covers most of Southwest China (Figure 1a). Based on the Global Forecast System (GFS) data provided by the National Centers for Environmental Prediction, observations including the Earth’s surface, radiosonde, radar, and satellite datasets were assimilated as the initial conditions and model boundary using an observation data assimilation analysis system [34,35]. GFS data were obtained for free every day, with high stability and data quality. By calculating meteorological elements including heat, moisture, humidity, and air flow velocity, the GFS provides the global atmospheric circulation situation and distribution of meteorological elements. In addition, the GFS forecasts future changes in atmospheric environment. The WRF model was initiated every day at 00:00 UTC to generate a 72 h forecast, which provided the meteorological data for the CMAQ model. The domain in the WRF model, which centered on 29.2 °N, 99 °E, had a horizontal resolution of 920 × 660 (~3 km) with 51 vertical levels. The WRF model initially decodes and preprocesses a wide range of observational data sources, including soundings, high altitude winds, aircraft reports, conventional ground stations, airport surfaces, ships, buoys, ground-based automated stations, radar, and satellites. The process of assimilation is subsequently conducted by employing an observational data assimilation analysis system to revise the initial fields of the WRF model. Following the formatting of the data and their vertical interpolation, predictions were generated utilizing the WRF integration module. Regarding the physical parameterization schemes, we applied the Yonsei University scheme (YSU) as the boundary layer scheme, the Thompson microphysical process scheme, the Noah land surface process scheme, the Fifth-Generation Penn State/NCAR Mesoscale Model scheme (MM5) as the near-surface process scheme, and a newer version of the Rapid Radiative Transfer Model scheme (RRTMG) as the shortwave and long wave radiation scheme. The cumulus parameterization scheme was closed. The YSU scheme enhances the depiction of the entrainment process by intensifying the mixing of thermally driven free convection and reducing the mixing of mechanically driven forced convection. On the other hand, the Thompson scheme is the pioneering microphysical process scheme that incorporates the impacts of aerosols, making it well-suited for high-resolution numerical simulation investigations. The RRTMG radiation scheme prioritizes the examination of aerosol impacts on the atmosphere. Consequently, incorporating the RRTMG radiation scheme enables the consideration of pollution effects on the atmosphere, thereby enhancing the simulation’s fidelity to real-world conditions.

In the CMAQ model, the forecast area covered the Sichuan Basin (Figure 1a). We used the Multi-resolution Emission Inventory model for Climate and air pollution research (MEIC) model developed by Tsinghua University, which includes industrial, civil, agricultural, and traffic pollutant sources, and also considers major atmospheric air pollutants (e.g., PM_2.5_, PM_10_, SO_2_, NO*_X_*, etc.) [36,37]. In the CMAQ model, the forecast concentration for the preceding day (beginning at 00:00 UTC) was used as the initial condition, and the vertical chemical profile file (i.e., bcon_profile) was used as the boundary condition in the chemical model. The CMAQ model was initiated from 00:00 UTC every day to perform a 72 h forecast. The domain in the CMAQ model had a horizontal resolution of 350 × 300 (~3 km) with 16 vertical levels. Finally, the cb05tucl_ae6_aq setting for the chemical mechanism and aero6 setting for the aerosol mechanism were selected.

### 2.3. Forecast Evaluation Method and Dataset

The autumn–winter period is defined as November through the following February. Four autumn–winter periods are included in the analysis period of 2018–2022 (i.e., November–February in 2018–2019, 2019–2020, 2020–2021, and 2021–2022); hereafter, this period is referred to as autumn–winter in 2018–2022.

The air quality index (AQI) derived from the China National Environmental Monitoring Centre (CNEMC) and daily PM_2.5_ concentration in Chengdu during autumn–winter in 2018–2022 were used for pollutant monitoring. The daily PM_2.5_ concentration, which was output from the CMAQ model and averaged over the seven environmental monitoring stations in Chengdu (red dots, Figure 1b) from 1 November 2021 to 28 February 2022, was the forecasted air pollutant.

Daily 2 m temperature, 2 m relative humidity, and 10 m wind fields averaged over the 14 meteorological stations in Chengdu (dark green dots, Figure 1b) from 1 November 2021 to 28 February 2022 were used as the meteorological observation values.

The outputs from the WRF model were first processed using the Meteorology–Chemistry Interface Processor (MCIP) weather model in the CMAQ model. Then, the mean daily 2 m temperature, 2 m relative humidity, and 10 m wind fields over the 14 meteorological stations in Chengdu were calculated as the forecasted meteorological factors.

The accuracies of the forecasts were evaluated using the following equation:

(1) $$R=\frac{\sum_{i=1}^{N}\left(O_{i}-\bar{O}\right)\left(P_{i}-\bar{P}\right)}{\sqrt{\sum_{i=1}^{N}{\left(O_{i}-\bar{O}\right)}^{2}}\sqrt{\sum_{i=1}^{N}{\left(P_{i}-\bar{P}\right)}^{2}}}$$

where R is the correlation coefficient between the observation and forecast time series; $P_{i}$ and $O_{i}$ are the ith forecast and observation, respectively; $\bar{P}$ and $\bar{O}$ are the average of all forecasts and observations, respectively; and N is the ensemble number. R values closer to 1 indicate a more accurate forecast [38].

## 3. Results

### 3.1. Objective Synoptic Weather Classification

**Figure 2:**
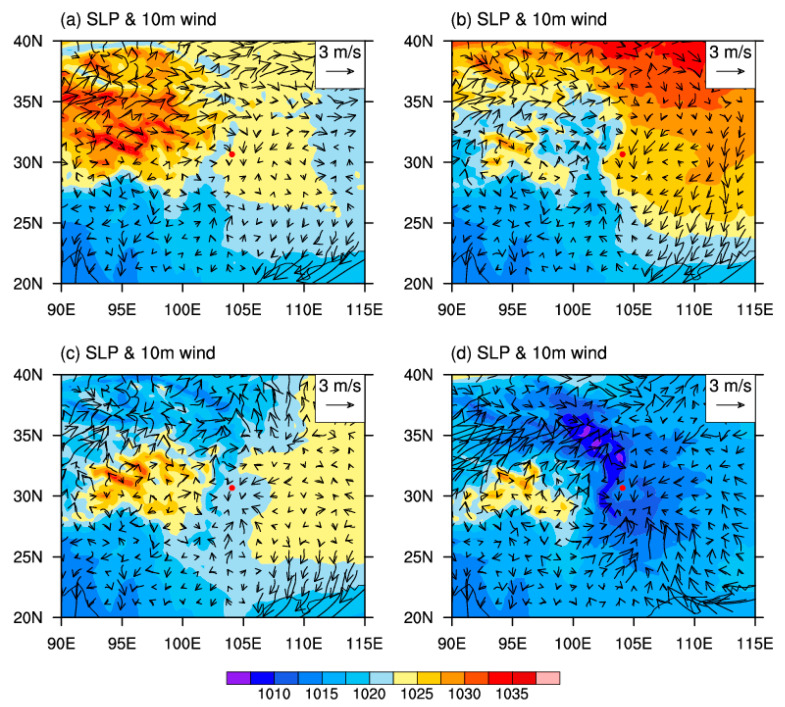
Spatial distributions of sea level pressure (SLP; hPa) and 10 m wind fields (m/s) in Chengdu during autumn–winter in 2018–2022 under the four dominant weather patterns: (**a**) high-pressure rear; (**b**) high-pressure bottom; (**c**) homogeneous-pressure; and (**d**) low-pressure conditions.

Figure 2 presents the four weather patterns derived from the objective synoptic weather classification method using the ERA5 product during autumn–winter in Chengdu: high-pressure rear, high-pressure bottom, homogeneous-pressure, and low-pressure conditions. The total variance explained by all weather patterns exceeds 85%. Under high-pressure rear conditions (Figure 2a), the Sichuan Basin, northern Shanxi Province, and Chongqing are controlled by a high-pressure center with a maximum value of 1025 hPa; Chengdu, which is located at the rear of this high-pressure center, is dominated by northerly and weak southerly winds with a speed of ~1 m/s. Under high-pressure bottom conditions (Figure 2b) in Gansu and Shanxi provinces, the high-pressure center has a maximum value of 1035 hPa. At the bottom of the high-pressure center, Chengdu is dominated by easterly winds with speeds of 1.5–3.0 m/s. Homogeneous-pressure conditions (Figure 2c) are characterized by homogeneous pressure of around 1022.5 hPa in Chengdu, in which northerly winds with an amplitude of ~1 m/s prevail. Finally, under low-pressure conditions (Figure 2d), low pressures (minimum of 1005 hPa) occur in the Sichuan Basin, Gansu, Shanxi, and Guizhou provinces, and Chongqing, and Chengdu is affected by northerly winds with a speed of ~1 m/s.

Under all four weather patterns, Chengdu has areas with relatively low-pressure centers, indicating that it is dominated by cyclonic convergence. Cyclonic convergence induces the advection of air pollutants around Chengdu toward the city center and leads to the upward transportation of air pollutants due to the cyclone-induced pumping effect. Meanwhile, a cold, high-pressure pattern controls northern Sichuan Province, generating northerly winds. These winds transport cold air to Chengdu, which facilitates the diffusion of air pollutants. Due to weak pressure gradients, the wind fields in Chengdu are relatively small under the high-pressure rear, homogeneous-pressure, and low-pressure conditions; as a result, Chengdu is prone to air pollution.

### 3.2. Correlation Analysis of Objective Weather Classifications and Fine Particle Concentrations

Table 1 presents the cumulative occurrence of each weather pattern, number of days and rate of fine particle concentrations exceeding the standard, and fine particle concentrations. The occurrence of weather patterns followed the decreasing order of high-pressure bottom (78.4%), high-pressure rear (12.5%), homogeneous-pressure (7.5%), and low-pressure (1.7%) conditions. In total, 142 days (29.5%) had fine particle concentrations above the air quality standard. The proportions of above-standard fine particle concentrations for each of the four weather patterns were consistent with the weather pattern proportions, and followed the decreasing order of low-pressure (87.5%), homogeneous-pressure (52.8%), high-pressure rear (35.0%), and high-pressure bottom (25.2%) conditions.

In terms of the occurrence rate of above-standard fine particle concentrations, the low-pressure pattern had the highest rate (87.5%), followed by the homogeneous-pressure pattern (52.8%); by contrast, the high-pressure rear and bottom patterns had occurrence rates below 50%. Under low-pressure and homogeneous-pressure conditions, Chengdu experiences low pressures with relatively small pressure gradients; this results in weak wind fields that limit the capacity for air pollutant diffusion. This explained the high occurrence rates of above-standard fine particle concentrations under low-pressure and homogeneous-pressure conditions. By contrast, under high-pressure bottom conditions, Chengdu is governed by cold air from the north, which increases pressure gradients, strengthens wind speeds, and enhances the diffusion capacity of air pollutants; thus, this weather pattern is associated with the lowest rate of above-standard fine particle concentrations. Overall, fine particle pollution is more likely to occur under low-pressure, homogeneous-pressure, and high-pressure rear conditions than under high-pressure bottom conditions.

### 3.3. Forecast Accuracy for Objective Weather Classifications and Fine Particle Pollution

#### 3.3.1. Forecast Accuracy for Objective Weather Classifications

Based on the objective synoptic weather classification using the fine particle concentrations provided by the CNEMC, we evaluated the 24, 48, and 72 h forecasts of the CMAQ model for each weather pattern and fine particle concentrations. However, under the low-pressure conditions, there is only 1 day with above-standard fine particle concentrations; due to this low sample size, this pattern could not be further analyzed.

Under high-pressure bottom conditions, the R values between the forecasted and monitored fine particle concentrations are 0.67, 0.66, and 0.55 for the 24, 48, and 72 h forecasts, respectively (Table 2), which are greater than those under high-pressure rear and homogeneous-pressure conditions. Overall, the correlations between monitored and forecasted fine particle concentrations are greater for the 24 h forecast than for the 48 or 72 h forecasts.

**Figure 3:**
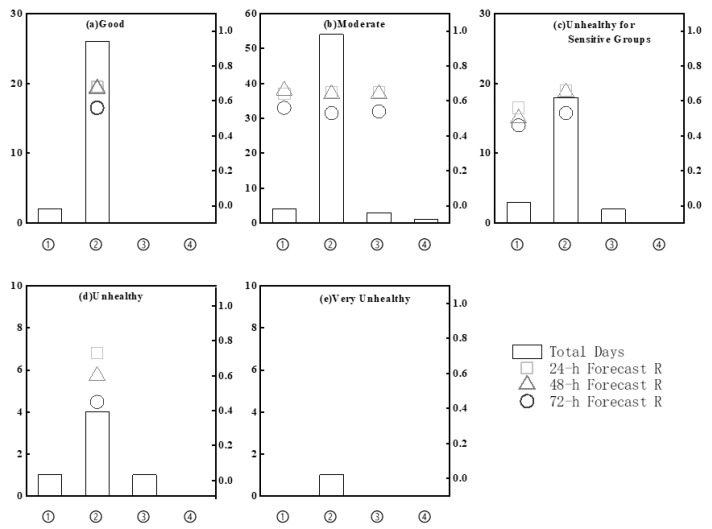
Evaluations of the 24, 48, and 72 h forecasts of daily fine particle concentrations in Chengdu during the period of 1 November 2021 to 28 February 2022 under the four dominant weather patterns (① high-pressure rear, ② high-pressure bottom, ③ homogeneous pressure, and ④ low pressure) based on the AQI: (**a**) good; (**b**) moderate; (**c**) unhealthy for sensitive groups; (**d**) unhealthy; and (**e**) very unhealthy.

To further evaluate the ability of the CMAQ model, we assessed the 24, 48, and 72 h forecasts of daily fine particle concentrations in Chengdu categorized by AQI under each weather pattern for the period of 1 November 2021 to 28 February 2022 (Figure 3). The percentages of days with good, moderate, unhealthy for sensitive groups, unhealthy, and very unhealthy are 23.3%, 51.7%, 19.2%, 5%, and 0.8%, respectively. Under high-pressure bottom conditions, the CMAQ model most accurately forecasted good, moderate, unhealthy for sensitive groups, and unhealthy AQIs for the 24 h forecast (R = 0.68, 0.65, 0.66, and 0.73, respectively), compared with the 48 and 72 h forecasts. Under high-pressure rear conditions, the CMAQ model most accurately forecasted moderate AQIs for the 48 h forecast (R = 0.66) and unhealthy for sensitive groups AQIs for the 24 h forecast (R = 0.56). Under homogeneous-pressure conditions, the CMAQ model most accurately forecasted moderate AQIs for the 24 h forecast (R = 0.65); however, this weather pattern lasted only 1–2 days; otherwise, the AQIs were good or unhealthy. Modeling of homogeneous-pressure conditions yields similar results with respect to unhealthy and unhealthy for sensitive groups. Due to the small sample sizes, the forecast accuracies under high-pressure rear and homogeneous-pressure conditions could not be further analyzed.

Overall, according to the AQI-based analysis, the model more accurately forecasted fine particle concentrations under high-pressure bottom conditions than high-pressure rear conditions. Moreover, 24 h forecasts were more accurate than 48 and 72 h forecasts. However, the AQI-based forecasts under each weather pattern have discrepancies, possibly resulting from our focus on daily pollution without accounting for pollution processes. For instance, assuming that fine particles could not readily accumulate on the first day of a weather pattern (i.e., a good AQI), the modeled air quality tends to deteriorate to unhealthy for sensitive groups on the second day; however, this trend differs from the observed results, which tend toward a moderate AQI on the second day. This difference may be attributable to the accumulated fine particle concentrations in the CMAQ model.

#### 3.3.2. Effects of Meteorological Factors on Fine Particle Forecast Accuracy

We examined the potential influence of hourly 2 m temperature, 2 m relative humidity, and 10 m wind speed predictions on the prognostication of fine particle concentrations within each weather pattern for 24, 48, and 72 h forecasts (Table 3). The 24 h forecasts of 2 m relative humidity (R = 0.64) and 10 m wind speed (R = 0.73) are more accurate under high-pressure bottom conditions than high-pressure rear or homogeneous-pressure conditions. However, no distinct differences are observed in the forecasts of 2 m temperature among the weather patterns. Considering that the forecasts of daily fine particle concentrations are more accurate under high-pressure bottom conditions than high-pressure rear or homogeneous-pressure conditions, 2 m relative humidity and 10 m wind speed likely influence the forecasting of fine particle concentrations. Under all weather patterns, the 24 h forecasts of the investigated meteorological factors are better than the 48 and 72 h forecasts, consistent with the fine particle concentration results.

## 4. Conclusions

In the present study, we analyzed the forecasting accuracy of fine particle concentrations in Chengdu during autumn–winter under four dominant weather patterns derived from COST733 objective weather classifications and the CMAQ model. Our main conclusions are summarized as follows.

During the autumn–winter period in Chengdu, fine particle pollution tends to occur under high-pressure rear, homogenous-pressure, and low-pressure conditions, and does not readily occur under high-pressure bottom conditions.

The daily fine particle concentrations under high-pressure bottom conditions are better forecasted in the 24 h forecast than in the 48 and 72 h forecasts. Similarly, the 24 h forecasts are better than the 48 and 72 h forecasts under the other weather patterns.

The 24 h forecasts of 2 m relative humidity and 10 m wind speed are more accurate under high-pressure bottom conditions compared to high-pressure rear and homogeneous-pressure conditions. Whereas 2 m temperature has no impact on the forecasting of fine particle concentrations, 2 m relative humidity and 10 m wind influence the fine particle concentration forecasts. Under all weather patterns, the 24 h forecasts of the meteorological factors are better than those of the 48 and 72 h forecasts, consistent with the fine particle concentration forecasts.

## Figures and Tables

**Table 1:** Cumulative occurrence of each of the four dominant weather patterns, number of days and rate of fine particle concentrations exceeding the standard, and range of fine particle concentrations exceeding the standard in Chengdu during autumn–winter in 2018–2022.

Weather Pattern	Cumulative Occurrence (Days)	Number of Days above the PM_2.5_ Standard (Days)	Occurrence Rate of PM_2.5_ Levels above the Standard (%)	Range of PM_2.5_ Concentrations above the Standard (μg/m^3^)
High-pressure rear	60	21	35.0	76–144
High-pressure bottom	377	95	25.2	76–186
Homogeneous pressure	36	19	52.8	76–131
Low pressure	8	7	87.5	77–155

**Table 2:** Evaluation of the 24, 48, and 72 h forecasts of daily fine particle concentrations under each of the four weather patterns, as well as the cumulative occurrence of each weather pattern, in Chengdu from 1 November 2021 to 28 February 2022.

Weather Pattern	Cumulative Occurrence (Days)	Forecast Duration	R Value of Predicted PM_2.5_
High-pressure rear	10	24 h	0.65
		48 h	0.65
		72 h	0.54
High-pressure bottom	103	24 h	0.67
		48 h	0.66
		72 h	0.55
Homogeneous pressure	6	24 h	0.65
		48 h	0.64
		72 h	0.54
Low pressure	1	24 h	-
		48 h	-
		72 h	-

**Table 3:** Evaluation of the 24, 48, and 72 h forecasts of temperature, relative humidity, and wind speed for each of the four weather patterns, as well as the cumulative occurrence of each weather pattern, in Chengdu from 1 November 2021 to 28 February 2022.

Weather Pattern	Cumulative Occurrence (Days)	Forecast Duration	R Value of 2 m Temperature	R Value of 2 m Relative Humidity	R Value of 10 m Wind Speed
High-pressure rear	10	24 h	0.95	0.63	0.73
		48 h	0.91	0.52	0.68
		72 h	0.89	0.53	0.66
High-pressure bottom	103	24 h	0.95	0.64	0.73
		48 h	0.92	0.51	0.67
		72 h	0.89	0.50	0.65
Homogeneous pressure	6	24 h	0.95	0.63	0.72
		48 h	0.91	0.50	0.67
		72 h	0.89	0.50	0.66
Low pressure	1	24 h	-	-	-
		48 h	-	-	-
		72 h	-	-	-

## Data Availability

The data that support the findings of this study are available upon reasonable request from the authors.
